# PSGL-1 Immune Checkpoint Inhibition for CD4^+^ T Cell Cancer Immunotherapy

**DOI:** 10.3389/fimmu.2021.636238

**Published:** 2021-02-23

**Authors:** Julia M. DeRogatis, Karla M. Viramontes, Emily N. Neubert, Roberto Tinoco

**Affiliations:** Department of Molecular Biology and Biochemistry, University of California, Irvine, Irvine, CA, United States

**Keywords:** PSGL-1, immune checkpoints, CD4^+^ T cells, anti-tumor immunity, cancer immunology

## Abstract

Immune checkpoint inhibition targeting T cells has shown tremendous promise in the treatment of many cancer types and are now standard therapies for patients. While standard therapies have focused on PD-1 and CTLA-4 blockade, additional immune checkpoints have shown promise in promoting anti-tumor immunity. PSGL-1, primarily known for its role in cellular migration, has also been shown to function as a negative regulator of CD4^+^ T cells in numerous disease settings including cancer. PSGL-1 is highly expressed on T cells and can engage numerous ligands that impact signaling pathways, which may modulate CD4^+^ T cell differentiation and function. PSGL-1 engagement in the tumor microenvironment may promote CD4^+^ T cell exhaustion pathways that favor tumor growth. Here we highlight that blocking the PSGL-1 pathway on CD4^+^ T cells may represent a new cancer therapy approach to eradicate tumors.

## Introduction

Early cancer treatments primarily relied on surgery, radiation, and chemotherapy. Newer therapeutic strategies were developed targeting oncogenic pathways, which extended the median overall survival of patients with advanced disease, but many eventually progressed due to tumor resistance and/or recurrence. Recent innovative therapeutic advances have now focused on harnessing and reinvigorating the immune response against tumors to provide novel treatment for cancer patients. Importantly, many of these advances have led to increases in median overall survival and sustained progression-free survival, especially for previously terminal cancer types such as metastatic melanoma. While new technologies such as CAR T cells have had significant success in non-solid cancers, immune checkpoint therapies using biologics have shown success against solid tumors. In addition, adoptive cell therapies which transfer expanded tumor-infiltrating T cells, along with dendritic cell cancer vaccines, have shown efficacy in various tumor types. The injection of exogenous cytokines such as IL-2 and IL-12 has also been successful at promoting anti-tumor immunity. Most of these strategies have focused on stimulating CD8^+^ cytotoxic T lymphocytes (CTLs), which are the main T cell populations that kill tumors. Even though CTLs are required for anti-tumor immunity, CD4^+^ T cells are also at the forefront of improving immunotherapies through their ability to provide help and amplify the CTLs response, making these cells an important target for cancer immunotherapy.

In the tumor microenvironment (TME), CD4^+^ T cells can directly kill tumors and/or change the TME to promote anti-tumor immunity (1, 2). CD4^+^ T cell help is now recognized to occur through a secondary priming step, where both CD4^+^ and CD8^+^ T cells recognize their cognate antigen on the same dendritic cell leading to optimal CTL clonal expansion and activation (3–8). This key CD4^+^ T cell help occurs through the licensing of dendritic cells (cDC1) through their interaction with MHC II and CD40/CD40L on CD4^+^ T cells, which was recently demonstrated *in vivo* in tumors (9), and resulted in successful cross presentation of tumor antigens to CD8^+^ T cells. Dendritic cell maturation and stimulation *via* CD40/CD40L by CD4^+^ T cells leads to IL-12, IL-15, and type I interferon production, which support CTL differentiation and function (10–16). Mature cDC1 cells also increase expression of MHC-I, CD80, and CD86 which provide activating and co-stimulatory signals to CD8^+^ T cells to promote their function (11, 14). Further, CD4^+^ T cell licensed cDC1 cells upregulate CD70 which can engage CD27 on CD8^+^ T cells to upregulate IL-12R and respond to IL-12 cytokines (17, 18). Activated CD4^+^ T cells also produce IL-2 and IL-21, which support differentiation, proliferation, and survival of CD8^+^ T cells (19, 20). These functions by CD4^+^ T cell-induced changes on dendritic cells are key in shaping the cross-priming required for effective activation of anti-tumor CD8^+^ T cells.

The specific ways through which CD4^+^ T cells help CD8^+^ T cell effectors have been investigated in numerous studies. In mice, key transcriptional changes in CD8^+^ T cells that differentiate under conditions with supportive CD4^+^ T cell help have been reported. These include the expression of the transcription factors T-BET, EOMES, and ID3, which regulate CTL differentiation (21–23). In addition, CTLs produce effector molecules such as TNF-α, IFN-γ, and granzyme B (GZMB) which promote their killing capacity and IL-2, which supports their cell-intrinsic differentiation and survival (17, 24). CTLs also express matrix metalloproteinases and upregulate expression of CX_3_CR1 and CXCR4, which enhance their migration properties (17). CD4^+^ T cell help also induces the downregulation of the immune checkpoints TIM3, BTLA, LAG3, and PD-1 which may prevent an exhaustion phenotype in CTLs in tumors (17).

While CD4^+^ T cells support CTLs, these cells also have direct anti-tumor activity and can kill tumors through engaging p-MHC II and secretion of IFN-γ and TNF-α effector cytokines (1, 2). Indeed, class II tumor-associated antigens have been identified including mucin 1, tyrosinase, premelanosome protein gp100, survivin, telomerase reverse transcriptase, and many others (25). CD4^+^ T cells can also provide help to B cells *via* CD40/CD40L and p-MHC II/TCR interactions which drive antibody responses to tumor-associated antigens (26, 27). Cancer vaccines that target both MHC class I and MHC class II epitopes were shown to bypass immune tolerance to induce effective antitumor immunity (28, 29). These studies highlight the importance of the CD4^+^ T cell response in anti-tumor immunity.

Effective T cell activation requires multiple signals including TCR-p-MHC, co-stimulation, and cytokines by antigen presenting cells that promote T cell proliferation and survival (6). In addition to co-stimulatory signals, T cell inhibitory pathways such as PD-1/PD-L1 and CTLA-4 are also important as “breaks” that ensure effective TCR signaling required for their differentiation (30). Both CD4^+^ and CD8^+^ T cells express these immune checkpoints which are upregulated after activation and then downregulated after antigen clearance; however, during chronic antigen stimulation such as in tumors and chronic viral infections, the expression of immune checkpoints remains elevated to promote the generation of exhausted CD4^+^ and CD8^+^ T cells (31, 32). Immune checkpoints inhibit T cells through multiple mechanisms, but their main function is to inhibit TCR signaling (31, 32). PD-1 signaling for example, has been reported to interfere with both TCR and CD28 signaling pathways to limit T cell activation (33). Many additional immune checkpoints including TIGIT, LAG-3, TIM-3, VISTA, CD160, and BTLA have been discovered and work through diverse mechanisms to limit T cell activation and these have been reviewed elsewhere (31, 34). These immune checkpoints are therapeutically significant, as antibody blockade of these surface expressed proteins have now been used to reinvigorate T cells in tumors to promote tumor control.

Clinical success targeting PD-1 and CTLA-4 has shown the most promise in promoting long-term durable responses in melanoma cancer patients (35). Importantly, these immune checkpoint blockade therapies have shown clinical efficacy in the treatment of multiple cancer types including melanoma, lung, kidney, and certain colon cancers, with many clinical trials currently ongoing (36, 37). Furthermore, these immune checkpoint inhibitors are in clinical trials to determine efficacy in additional cancers (37). While these therapies show efficacy as monotherapies, greater responses are observed when these immune checkpoint inhibitors are combined (38). In addition, combining radiation and chemotherapy with immune checkpoint inhibitors have also shown clinical efficacy (39, 40), supporting the notion that combination therapies can be more beneficial than these treatments alone. Despite the clinical success using immune checkpoint inhibitors, 50–80% of patients receiving these therapies fail to respond to treatment (41). In addition, immune-related adverse events (irAEs) are observed in patients receiving these treatments with some reported deaths (35). It is clear that a better understanding of resistance mechanisms to these therapies are needed to improve cancer patient outcomes while limiting irAEs. It is also important to develop strategies that target new immune checkpoints that can improve anti-tumor immunity as either monotherapies or combination therapies. A new immune checkpoint, P-selectin glycoprotein ligand-1 (PSGL-1, *Selplg* gene) has been shown to inhibit anti-tumor responses in pre-clinical models and may represent a new strategy to improve patient outcomes (42). While much focus has centered on improving CTL responses, the fact that many patients are unresponsive to current immune checkpoint blockade therapies highlight that CD4^+^ T cell help may be defective in these patients, especially since immune checkpoint inhibitors also reinvigorate CD4^+^ T cell functions. PSGL-1 deficient (*Selplg^−/−^*) mice were shown to have enhanced anti-viral and anti-tumor immunity, with T cells escaping functional exhaustion partly due to their enhanced CD4^+^ T cell responses (42). These findings, together with prior work showing PSGL-1 functioned as a negative regulator in T cells (43), suggest that inhibiting PSGL-1 in CD4^+^ T cells through multiple immunotherapeutic strategies may represent a new strategy to improve anti-tumor immunity.

## CD4^+^ T Cells Express PSGL-1

The initial studies of PSGL-1 focused primarily on its expression and role in neutrophils, but PSGL-1 has since been identified on all myeloid and lymphoid lineages. Despite the ubiquitous expression of PSGL-1 on all hematopoietic lineages, its expression level and functionality differ among these cell types. On myeloid cells, PSGL-1 is constitutively expressed in its functional form, which has the posttranslational modifications required for selectin binding (44). Within the lymphocyte lineage, all T cell subsets express PSGL-1, whereas very low to undetectable expression is present on B cells (45). Similar to myeloid cells, T cell binding and endothelial migration is regulated by PSGL-1. Unlike myeloid cells, however, T cells do not constitutively express functional PSGL-1 and must express the enzymatic machinery required to modify PSGL-1 during T cell activation (44). Although PSGL-1 is expressed on all T cell subsets, including both Th1 and Th2 CD4^+^ T cells, Th2 cells do not express the functional form and thus have decreased binding capacity to P- and E-selectins when compared to Th1 cells (46). In follicular T helper cells (TFH), PSGL-1 downregulation by the transcription factors Bcl6 and Ascl2 facilitates migration in follicles (47). Another CD4^+^ T cell subset with expression of highly functional PSGL-1 are Tregs, which have been explored in a model of experimental autoimmune encephalomyelitis (EAE), where PSGL-1 expression was linked to the suppressive capacity of Tregs (48). PSGL-1 is also expressed by Th17 and CD8^+^ T cells (49).

## Tumor Immune Response in PSGL-1-Deficient Mice

Preclinical studies in WT and *Selplg^−/−^* mice showed that mice injected with melanoma cell lines (YUMM1.5) developed subcutaneous tumors, however *Selplg^−/−^* mice had increased infiltration of effector CD4^+^ and CD8^+^ T cells. *Selplg^−/−^* T cells also had increased production of IFN-γ, TNF-α, and IL-2 which led to melanoma tumor control (42). Furthermore, CD4^+^ and CD8^+^ T cells in *Selplg^−/−^* mice had decreased PD-1 expression, indicating that these cells were phenotypically less exhausted than WT cells (42). CD4^+^ T cell-derived IL-2 would support the concept that *Selplg^−/−^* CD4^+^ T cells provided enhanced helper function to CTLs than WT cells, which led to complete responses in ~18% of *Selplg^−/−^* mice, although this was not evaluated (42). Even though Tregs numbers were similar in tumors in WT and *Selplg^−/−^* mice, the ratio of effector T cells to Tregs was increased in *Selplg^−/−^* mice (42). These studies also showed that while PSGL-1 is important in T cell migration, PSGL-1 was not required for their entry in tumors, as shown by the highly infiltrated tumors in *Selplg^−/−^* mice compared to WT. Adoptive cell transfer of TCR transgenic OT-I CD8^+^ T cells into B16-Ova melanoma tumor bearing mice also showed that *Selplg^−/−^* OT-I T cells were superior at promoting tumor killing than WT cells (42). Interestingly, others examined the ability of *Selplg^−/−^* to control B16 melanoma and did not observe enhanced B16 tumor control and tumors were larger (50). These contrasting findings highlight that *Selplg^−/−^* mice may elicit different immune responses in various tumor models, and in the case of B16, may only be controlled through the use of adoptive cell therapy of previously activated T cells.

## Human and Mouse PSGL-1

**Figure 1:**
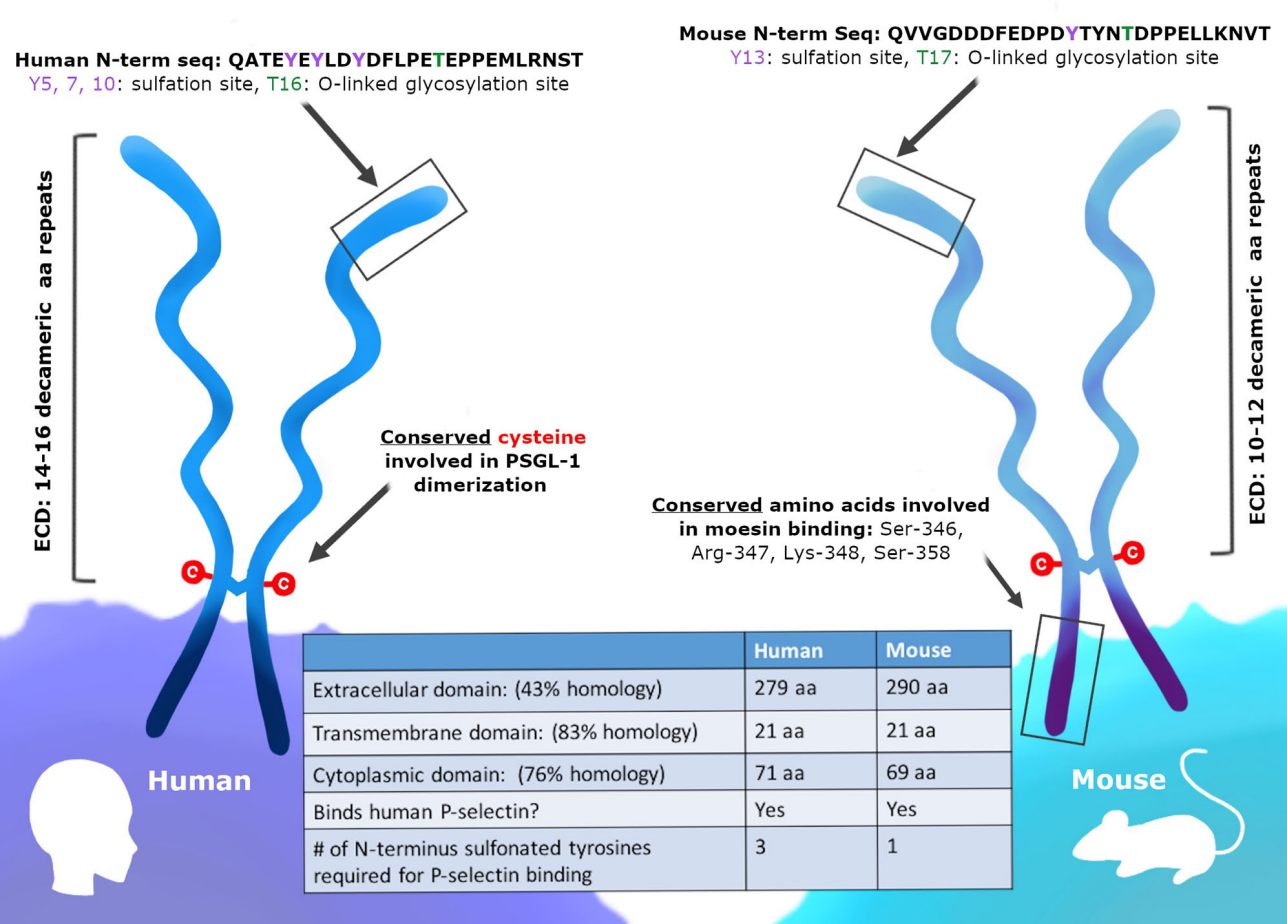
PSGL-1 is expressed in mice and humans. PSGL-1 is expressed as a homodimer on the surface of most hematopoietic cells. Similarities and differences between mouse and human PSGL-1 are shown.

While humans and mice both express PSGL-1, it is important to consider the similarities and differences between these two proteins (**Figure 1**). Murine PSGL-1 is encoded as a 397 amino acid (a.a.) protein (51). The mature form of murine PSGL-1 has a 290 aa extracellular domain (ECD), which contains 10 decameric repeats. In contrast, human PSGL-1 is encoded as a 412 aa protein and has a 279 aa ECD that contains 16 decameric repeats (52). The sequence of the decameric repeats also differs between human and murine PSGL-1. The human consensus sequence is -A-T/M-E-A-Q-T-T-X-P/L-A/T-, while the murine consensus sequence has been described as -E-T-S-Q/K-P-A-P-T/M-E-A- (51–53). Studies comparing the amino acid sequence of human and murine PSGL-1 have found the two proteins only share 43% sequence similarity in the ECD, although the transmembrane and cytoplasmic domains are more similar (51). While the sequences may be different, murine and human PSGL-1 share important similarities in the regions of the protein that are involved in ligand binding and signaling. In order to interact with selectins, the N-terminus of PSGL-1 must undergo core-2 O-glycosylation of a threonine and sulfation at tyrosine residues (53). In murine PSGL-1, O-glycosylation occurs at Thr17 and only one tyrosine is sulfated, Tyr13 (54). In human PSGL-1, the O-glycosylation occurs at Thr16 and there are three sites of tyrosine sulfation (Tyr5, 7, and 10) instead of one (**Figure 1**). In both human and mice, PSGL-1 has a cysteine residue that precedes the transmembrane domain and facilitates dimerization, as well as conservation of serine, lysine, and arginine residues in the cytoplasmic moesin-binding sequence (55). Additionally, an aspartic acid, a lysine, and a valine are conserved between species in the versican-binding region of the protein (55, 56). While more research is needed into the signaling differences between human and murine PSGL-1, it is clear that the selectin-binding function of the protein is conserved, as well as the types of post-translational modifications that occur at the N-terminus (**Figure 1**).

When considering the translatability of mouse PSGL-1 studies, the differences between mouse and human PSGL-1 biology must be understood. While the selectin-binding function of the N-terminus of murine and human of PSGL-1 is conserved, the differential requirements for P-selectin binding are important to note. As mentioned above, human PSGL-1 requires a core-2 O-glycan plus three sulfated tyrosine residues to bind P-selectin, of which two sulfated residues form direct bonds to the lectin domain (57–59). In contrast, the binding of murine PSGL-1 to P-selectin is facilitated largely by a core-2 O-glycan and a single sulfated tyrosine (54). When the canonically glycosylated threonine residues were mutated in human and murine PSGL-1, only human PSGL-1 binding to P-selectin was abolished, indicating that murine PSGL-1 does not depend on these glycosylated residues for binding. These studies highlight the differential contributions of post-translational modifications surrounding protein structure to the selectin binding ability of human and murine PSGL-1. These differences are important to understand, as targeting N-terminal residues or post-translational modifications on PSGL-1 may have different outcomes in humans and mice. The differences in the ligands that bind PSGL-1 should also be noted. While human PSGL-1 can bind Siglec-5, this ligand is not present in mice, and therefore may contribute to a phenotype in human studies not seen in mice (60). The possible ligand-receptor pairs can also differ from mice to humans, and these interactions may change depending on the immune cells and the microenvironments in which are they located. In a murine AML cell line, only PSGL-1 was capable of binding E-selectin (61). However, in human AML cells lines, both CD44 and PSGL-1 could bind E-selectin. The differences in PSGL-1 between species are important to consider, especially when these findings are applied for translational purposes such as therapeutic immune modulation.

## PSGL-1 Ligands

### Selectins

**Figure 2:**
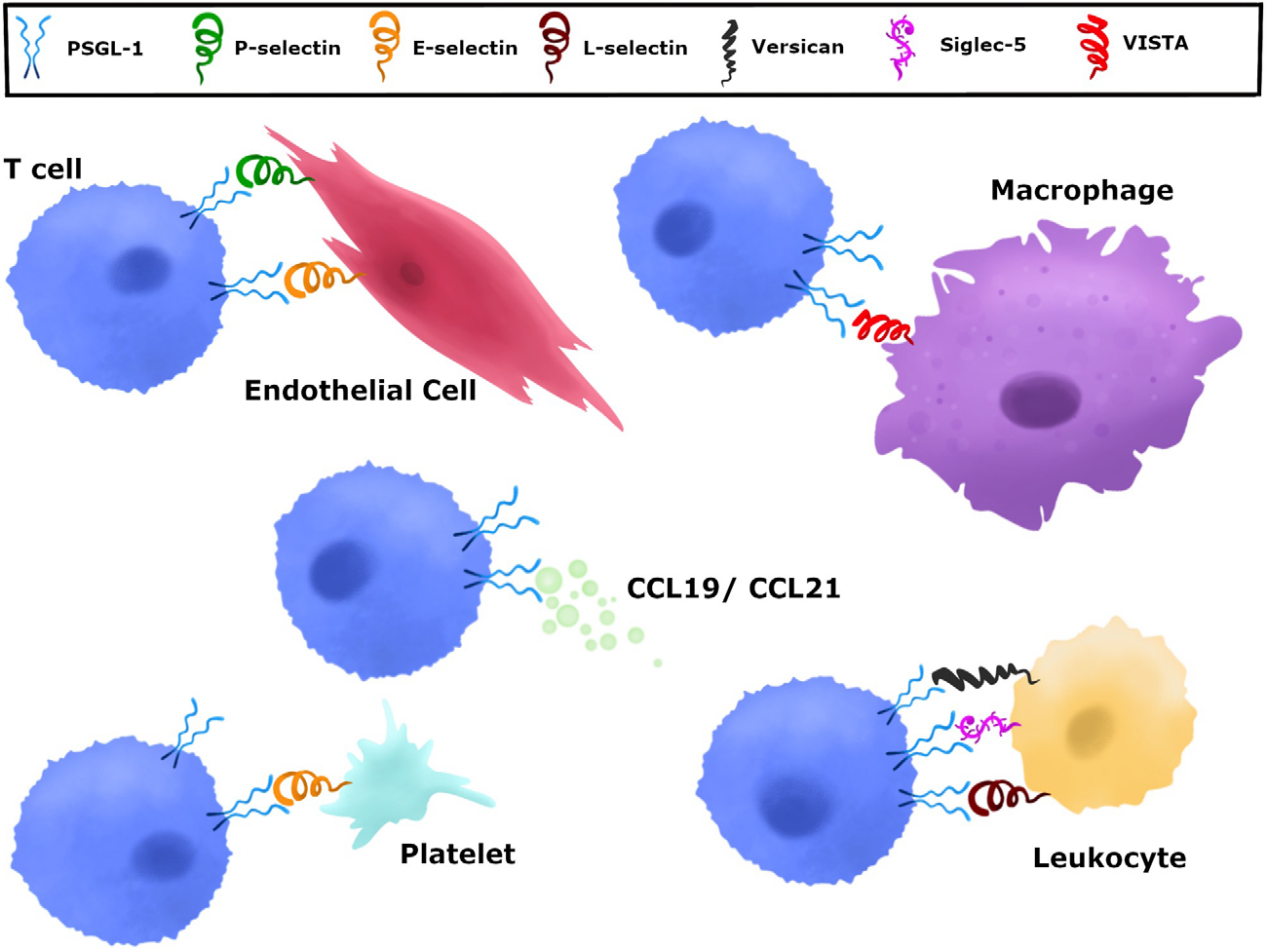
Various ligands can engage PSGL-1. PSGL-1 can bind P-, E-, and L-selectin. P-selectins are present in a variety of cells including platelets and endothelial cells. E-selectins are expressed by endothelial cells and L-selectin by leukocytes. CCL19 and CCL21 chemokines are present in secondary lymphoid organs and can be produced by endothelial cells, stromal cells, and mature dendritic cells. Versican is produced by epithelial, endothelial, stromal cells, and leukocytes. Siglec-5, which is only present in humans, is expressed in neutrophils, mast cells, monocytes, DCs, NK cells, and stimulated T cells. VISTA is expressed on myeloid cells and granulocytes.

Immune checkpoints have specific binding partners that inhibit T cells and PSGL-1 can engage a number of ligands. The mechanisms by which immune checkpoint receptors and ligands interact are key in understanding and developing strategies to reverse and/or prevent their inhibitory function. PSGL-1 proteins engage a diverse array of ligands at steady state and at different stages of the immune response. While multiple PSGL-1 ligands have been identified, the selectins were the first to be characterized and the most widely studied (51). All three selectins, platelet (P), endothelial (E), and leukocyte (L) have been well characterized to bind PSGL-1 through the N-terminus extracellular domain (54, 62, 63) (**Figure 2** and **Table 1**). However, the PSGL-1 binding affinities differ between the three, with P-selectin having the highest affinity, followed by E- and L-selectins, respectively (59, 64–66). Importantly, while all leukocytes can bind selectins due to PSGL-1 post-translational modifications (59, 67, 68), naïve CD4^+^ and CD8^+^ T cells engage selectins only after T cell activation (44). Naïve T cells express PSGL-1, however lack of sialylation and fucosylation on PSGL-1 prevent selectin binding (44). Various enzymes are involved in modifying PSGL-1 to allow P-selectin binding, including fucosyltransferase IV and VII, core 2 β1,6-glucosaminyltrasferase-I, β1,4-galactosyltransferase-I, sialyl 3-transferase IV, and tyrosylprotein sulfotransferase 1 or 2 (69). CD4^+^ and CD8^+^ T cell activation induces enzymatic activity which facilitate P-selectin binding (70–72). Furthermore, IL-12 signaling in Th1 cells was shown to induce PSGL-1 functionality, while IL-15 in CD8^+^ T cells induced core-2 O-glycan expression *in vitro* and *in vivo* (70, 73). Antigen-specific CD4^+^ T cells responding to influenza viral infection were shown to express functional PSGL-1 by 6 days post-infection (dpi) and memory T cells retained this post-translational modification to 30 dpi, with the majority of cells binding P-selectin in the lungs and draining lymph nodes (74). This *in vivo* study showed that P-selectin binding in CD4^+^ T cells occurs early during the immune response and persists to late timepoints after infection, when these cells differentiate to form memory T cells (74). These findings indicate that after naïve T cell activation, CD4^+^ T cells retain enzymatic activity that modifies PSGL-1 to continue engaging P-selectin as memory T cells (74). PSGL-1 binding of these selectins plays a major role in leukocyte migration and recruitment. PSGL-1 expressing leukocytes circulating in the blood attach to activated endothelium expressing P- and E- selectins. This PSGL-1-mediated attachment allows leukocytes traveling at high velocities to attach, roll and tether to the endothelium and transmigrate to sites of inflammation, infection, and tumors (75–78).

**Table 1:** PSGL-1 and its binding partners.

Molecule	Gene Name	Cells expressed
P-selectin glycoprotein ligand-1 (PSGL-1)	*Selplg*	CD4^+^ T cells, CD8^+^ T cells, Tregs, HSCs, DCs, neutrophils, monocytes, macrophages, most lymphocytes, and granulocytes
V-domain Ig suppressor of T cell activation (VISTA)	*Vsir*	Myeloid cells, granulocytes, and T cells
Platelet selectin (P-selectin)	*Selp*	Platelets and endothelial cells
Endothelial selectin (E-selectin)	*Sele*	Endothelial cells
Leukocyte selectin (L-selectin)	*Sell*	Granulocytes, monocytes, and most lymphocytes
Versican	*Vcan*	Epithelial, endothelial, stroma, and leukocytes
Sialic Acid Binding Ig Like lectin 5 (Siglec-5)	*SIGLEC*_5_ *(Human)*	Neutrophils, mast cells, monocytes, DCs, NK, and T cells
C-C motif chemokine ligand 19 (CCL19)	*Ccl19*	Stromal cells and mature DCs
C-C motif chemokine ligand 21 (CCL21)	*Ccl21a, Ccl21b, Ccl21c*	Lymphatic endothelial and stromal cells

Recently, it was shown that PSGL-1 expression on primary acute leukemia myeloblasts and lymphoblasts was a major ligand interacting with the endothelial P- and E-selectins (79). Understanding PSGL-1 regulation on these blood cancer cells and their binding to endothelial selectins may provide insight into whether these interactions facilitate invasion and metastasis into various organs to seed new tumors. Additionally, novel cancer therapeutics to target these adhesion interactions *via* PSGL-1 may be beneficial in inhibiting lymphoma and leukemia extravasation. Limiting PSGL-1 and selectin interactions may also promote improved T cell responses. CD4^+^ T cells would acquire their ability to bind selectins in the tumor-draining lymph node as they engage cDC1 dendritic cells presenting tumor antigens. Recent work has shown that cDC1 cells are required to effectively activate both CD4^+^ and CD8^+^ T cell responses to tumors (9). Whether PSGL-1 on T cells and/or cDC1 dendritic cells contributes to T cell activation is unknown, but studies show that P-selectin engagement on DCs can induce a tolerogenic phenotype that can suppress T cells (80). Selectin dampening of T cell activation through PSGL-1 signaling is a potential suppressive mechanism by dendritic cells in tumor draining lymph nodes and/or tumors. Once T cells exit lymph nodes and enter circulation, they can activate additional signaling pathways through PSGL-1/selectin interactions on endothelial cells, inducing cellular migration through cytoskeleton rearrangement (81). It is unknown whether these migration signaling pathways alter the function of anti-tumor T cells as they enter tumors. Whether anti-tumor CD4^+^ and CD8^+^ T cells engage selectins in tumors and whether these interactions contribute to T cell exhaustion is unknown. A chronic viral infection model however, showed that blocking P, E, and L selectins did not reverse anti-viral CD8^+^ T cell exhaustion (42), indicating that other possible PSGL-1 binding partners outside of selectins may promote T cell exhaustion.

### Chemokines

While the selectins have been well studied as receptors for PSGL-1 in immune cell trafficking during inflammation, the chemokines CCL19 and CCL21 have also been shown to bind PSGL-1 under steady state conditions (82–84) (**Figure 2** and **Table 1**). The interactions between PSGL-1 and these chemokines are important for homing of resting lymphocytes into secondary lymphoid tissues. Mature dendritic cells can produce and secrete CCL19, whereas CCL21 is secreted by endothelial cells in lymphatic vessels. Both CCL19 and CCL21 are produced and secreted by stromal cells in the spleen, lymph nodes, and the lumen of high endothelial venules (85). Naïve and memory T cells can all bind these chemokines through CCR7 interactions, which provide lymphocytes multiple opportunities to circulate through secondary lymphoid tissues and detect antigens presented by antigen presenting cells (86, 87). Importantly, as CCR7^+^ effector T cells progress to an exhausted state during viral infection, they downregulate CCR7 expression (86). This CCR7 downregulation is also observed in virus-specific CD8^+^ T cells during lymphocytic choriomeningitis virus (LCMV) infection (88). It is at these key stages when CD4^+^ and CD8^+^ T cells downregulate CCR7 where they may have PSGL-1 accessible to interact with CCL19 and CCL21 chemokines (**Figure 2**). These chemokines could impact anti-tumor T cell responses through PSGL-1 engagement in the tumor draining lymph nodes. Indeed, CCL19 and CCL21 have been shown to induce activation induced cell death (AICD) of responding CD4^+^ T cells (89). PSGL-1 engagement by these chemokines in tumor draining lymph nodes may induce cell death of anti-tumor T cells, resulting in decreased effector T cells exiting lymph nodes and thereby reducing infiltration in tumors. CD4^+^ and CD8^+^ T cells interactions with dendritic cells during priming and later stages of T cell activation could be meditated by PSGL-1 and CCL19/CCL21 interactions, since inflammatory dendritic cells also express PSGL-1 (90). Furthermore, whether cDC1 cells utilize PSGL-1 during tumor antigen presentation to both CD4^+^ and CD8^+^ T cells is unknown. Clearly, the interactions of PSGL-1 on immune cells with CCL19/CCL21 are under studied. More work is needed to provide insight into how PSGL-1/chemokine interactions and signals may be playing a role in the anti-tumor T cell response.

### Versican

Versican, a chondroitin sulfate proteoglycan that is found in the extracellular matrix of a wide range of cell types including epithelial, endothelial, stromal cells, and leukocytes has also been shown to bind PSGL-1 (56, 91, 92) (**Figure 2** and **Table 1**). Some of its functions include mediating cellular adhesion, migration, proliferation, and differentiation (93–96). The specific binding between PSGL-1 and versican has been reported to mediate leukocyte aggregation (56). In addition to binding PSGL-1, versican can also bind TLR2 and P- or L-selectin (56, 97–101) and is reported to be both pro- and anti-inflammatory. Mice treated with LPS and siRNA to inhibit versican showed increased leukocyte infiltration into the lungs and inflammatory TNF-α, NFkB, and TLR2 levels, illustrating that versican can limit inflammation (102). Macrophages stimulated with LPS showed an increase in versican expression as these cells became more inflammatory (103, 104). Versican is a relevant PSGL-1 ligand to consider during therapeutic design, as versican has been found to be increased in a number of cancers (105–107). In the tumor microenvironment, both cancer cells and stromal cells can be a source of versican (108–110). In the TME, myeloid cells produce versican and promote tumor metastasis and increased versican in tumors correlated with reduced CD8^+^ T cells infiltration (92, 111, 112). Tumor cell-derived versican can also induce the upregulation of PD-L1 on monocytes and macrophages (113), an important molecular driver of T cell exhaustion. As it is known that PSGL-1 binds versican, and that versican seems to be playing a pro-tumoral role in the TME, it is possible that versican-PSGL-1 interactions in the tumor microenvironment may inhibit T cell infiltration and prevent tumor killing (**Figure 2**). Versican is an important PSGL-1 ligand that should be investigated further and considered as a target for cancer immunotherapy.

### Siglec-5

Sialic acid-binding immunoglobulin-type lectins (Siglecs), are expressed on the cell surface of both innate and adaptive immune cells (114). These surface receptors recognize and bind glycans and are involved in various diseases including sepsis and cancer (115–118). While multiple Siglecs have been identified in humans and mice, Siglec-5 (only expressed in humans), has been shown to bind PSGL-1 (119) (**Figure 2** and **Table 1**). Siglec-5 is expressed in neutrophils, mast cells, monocytes, pDCs, *in vitro* generated DCs, NK cells, and in T cells after stimulation (120–125). PSGL-1 is highly sialylated and was found to bind soluble Siglec-5 in a calcium- and dose-dependent manner (119). Furthermore, sialidase treatment of PSGL-1 reduced Siglec-5 binding. Studies also showed that on human PBMCs, both Siglec-5 and PSGL-1 are closely associated, and *in vitro* perfusion assays demonstrated that soluble Siglec-5 inhibited leukocyte rolling on E- and P-selectin, indicating that Siglec-5 may have an anti-adhesive role. This was also observed in a model of TNF-α-induced inflammation, wherein injection of soluble Siglec-5 in mice prevented inflammatory leukocyte recruitment (119). While it appears that Siglec-5 may inhibit leukocyte migration, the contribution of PSGL-1 and Siglec-5 binding on anti-tumor T cells and role in anti-tumor immunity is unknown.

### VISTA

New ligands for PSGL-1 have recently emerged. V-domain immunoglobulin suppressor of T cell activation (VISTA) was shown to be a negative regulator of T cells (126). Myeloid and granulocytes are the primary VISTA-expressing cells, however, T cells express low levels (126–128). Recently, VISTA was reported to bind PSGL-1 and suppress T cell activity in acidic conditions *in vitro*, similar to those found in tumor microenvironments (129) (**Figure 2** and **Table 1**). P-selectin binding to PSGL-1 is dependent on sulfotyrosine and sialyl-Lewis X tetrasaccharide modifications (130), while VISTA binding depends on tyrosine sulfation but not sialyl-Lewis X modifications on PSGL-1 (129). Moreover, blocking PSGL-1/VISTA binding reversed VISTA-mediated immune suppression (129). The suppressive binding of VISTA to PSGL-1 in acidic conditions may be a potential tumor evasion strategy, highlighting both a new role for PSGL-1 in tumors and the possibility of targeting PSGL-1 and/or VISTA for future cancer immunotherapies.

## PSGL-1 and Cell Migration

The importance of PSGL-1 in leukocyte migration was first established in a mouse model with genetically deleted *Selplg* (131). PSGL-1 deficiency resulted in increased neutrophils in the blood, suggesting a decrease in neutrophil adherence and migration. Indeed, the importance of PSGL-1 in facilitating early migration of neutrophils to sites of inflammation was demonstrated in a peritonitis model in *Selplg^−/−^* mice, in which neutrophil migration to the peritoneal cavity was significantly decreased (76, 132). *In vitro* and *in vivo* studies have shown PSGL-1 to be important for promoting leukocyte rolling in response to inflammation (131, 132). Functional studies have determined that the amino terminal of PSGL-1 is involved in the recruitment of neutrophils to sites of inflammation, as seen in murine colitis and peritonitis models where blockade of the amino terminal of PSGL-1 resulted in decreased leukocyte migration to inflammatory sites (131–134). Selectin capture of leukocytes occurs through interactions with the amino terminal of PSGL-1 and facilitates migration to sites of inflammation. Although P-selectin has been repeatedly shown to be a major ligand for PSGL-1 on neutrophils, further research has demonstrated that PSGL-1 can also promote neutrophil tethering to E-selectin and L-selectin, allowing for some leukocyte migration even in the absence of P-selectin (133, 135, 136). Interestingly, the tethering role of E-selectin seems to differ among cell types, as eosinophils tether less efficiently to E-selectin than neutrophils do (137).

**Figure 3:**
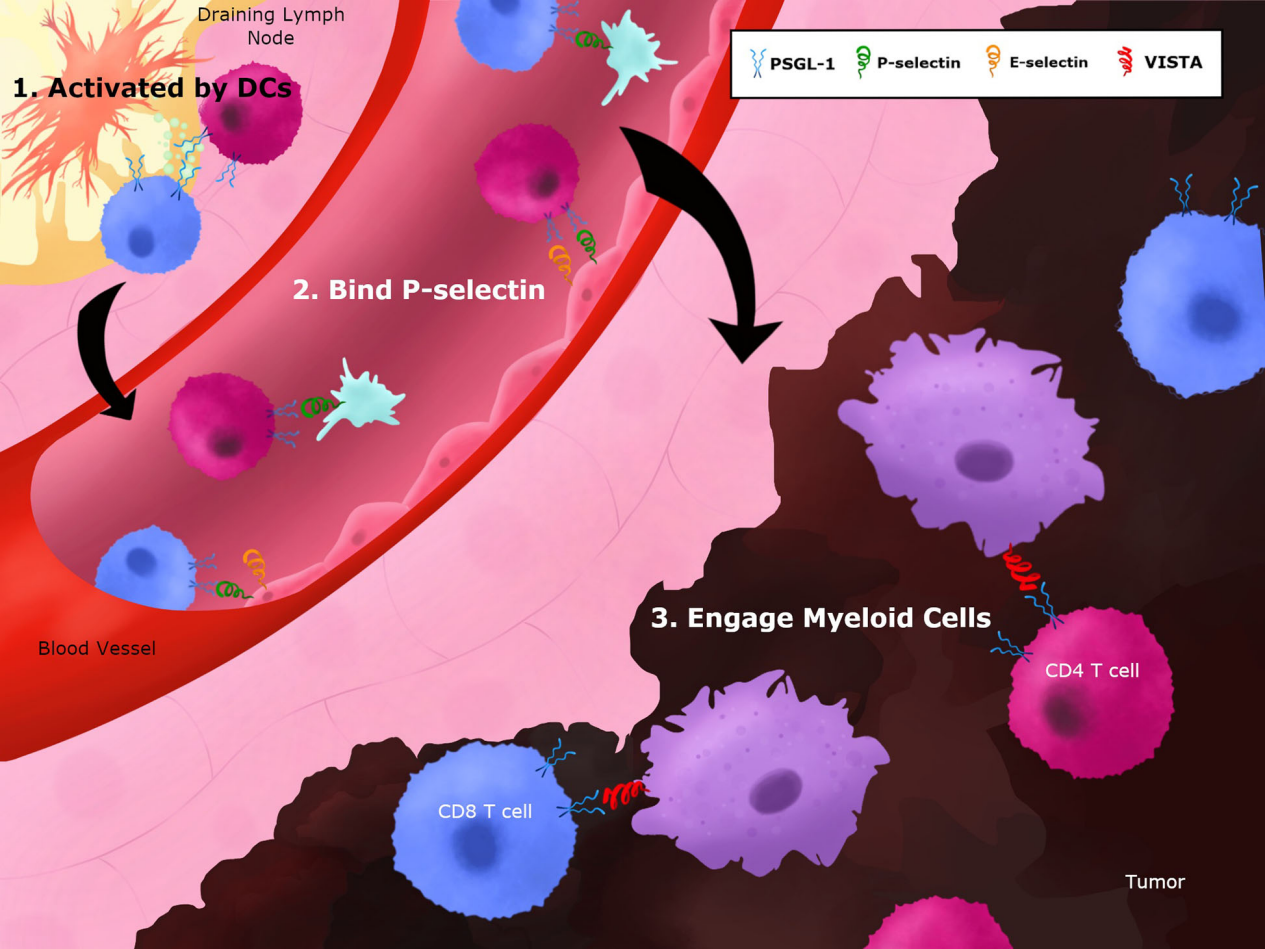
Potential PSGL-1 interactions on T cells throughout the course of an immune response. T cells migrate through various tissues in the course of a response to tumor antigens. **1**) In the tumor-draining lymph nodes, T cells can engage PSGL-1 during their priming and activation stage as they interact with mature dendritic cells. PSGL-1 can bind chemokines and/or L-selectins to facilitate priming and CD4^+^ T cell help to CTLs when they interact with cDC1 cells. **2**) T cells exit lymph nodes and enter the circulation where PSGL-1 can be engaged by P- or E-selectin, which induce signaling pathways while helping T cells to extravasate. **3**) T cells enter tumors and additional binding partners, such as VISTA, can bind PSGL-1 to promote T cell dysfunction in the TME.

While a large body of research has delved into the role of PSGL-1 on neutrophils, PSGL-1 is also expressed on all myeloid and most lymphoid cells, and has diverse roles in regulating migration and adhesion among these cell types, important when considering that many of these immune cells are present in the TME (**Figure 3**). T cell responses to tumors occur through the trafficking of these cells through various tissues including the tumor draining lymph node, the circulatory system, and the TME (**Figure 3**). These diverse tissues with diverse cell types provide opportunities by which PSGL-1 on T cells can be engaged by the available ligand(s) to modulate T cells (**Figure 3**). Multiple models of inflammation ranging from colitis and ileitis, to local skin damage, have shown that PSGL-1 can promote recruitment and phenotypic changes of DCs and macrophages, and can facilitate monocyte adhesion and rolling, as well as T cell migration (46, 75, 138–140). The importance of PSGL-1 in migration is evident by the fact that blocking the N-terminus of PSGL-1 with a monoclonal antibody (mAb) caused a substantial reduction in rolling and adhesion of leukocytes in the intestines of mice with inflammatory bowel disease (132, 139). The PSGL-1 mAb-treated mice in these studies tended to show improved disease outcome in colitis and ileitis, which is attributed to a reduction in the presence of inflammatory leukocytes. Interestingly, mice harboring a genetic PSGL-1 deletion had more inflammation and worse disease outcomes in dextran sodium sulfate or T cell-driven colitis (43, 75). These findings highlight that while PSGL-1 is involved in leukocyte migration, it is playing additional roles in immune modulation that can impact disease outcome.

The ways in which PSGL-1 directs T cell adhesion and movement are of particular interest, as an increased T cell infiltrate affects disease outcomes. On T cells, PSGL-1 mediates attachment and rolling on P- and E-selectin (140, 141). The binding of PSGL-1 to P-selectin on T cells was first demonstrated in an *in vitro* adhesion assay, and has since been shown numerous times *in vivo* (140, 142, 143). This binding of PSGL-1 to P- and E-selectin supports T cell migration into sites of inflammation, and has been demonstrated in both CD8^+^ and CD4^+^ T cells. It should be noted that the activation state of T cells affects PSGL-1-selectin interactions. Initial *in vitro* experimentation demonstrated that chronic stimulation of T cells led to increased rates of PSGL-1-P-selectin binding interactions (49). More recent studies have built upon this foundation, showing that in activated human T cells, PSGL-1 facilitates rolling on E-selectin, and that the marker of activation CD44 can work cooperatively with PSGL-1 to support activated T cell migration (144).

In addition to the functionality of PSGL-1 differing due to T cell activation state, PSGL-1 also has different roles depending on the subset of T cells. As mentioned above, PSGL-1 on CD4^+^ T cells acts as a ligand for P-selectin, as well as for E-selectin, and facilitates the migration of Th1 cells to inflamed skin (142). In contrast to Th1 cells, Th2 cells express a different form of PSGL-1 that is unable to mediate P-selectin binding and does not promote Th2 cell migration to the sites of inflammation (46). In this way, PSGL-1 not only directs immune cells toward inflammation but can alter the population dynamics of responding immune cells. As with Th1 cells, CD8^+^ T cell migration is directed in part by PSGL-1. Through the use of a murine model of contact hypersensitivity and *in vitro* generated type I cytotoxic T cells, it was found that PSGL-1 binding to E- and P-selectin assists in CD8^+^ T cell trafficking to inflamed skin sites (140). While it is clear that PSGL-1 binding to selectins drives effector T cell migration, the role of PSGL-1 on CD4^+^ and CD8^+^ T cells can differ. A study of isolated CD4^+^ T and CD8^+^ T cells from patients with multiple sclerosis revealed an expansion of the PSGL-1^+^CD4^+^ T cell population and linked PSGL-1 expression to the ability of CD4^+^ T cells to transmigrate blood-brain-barrier-derived endothelial cells (145). In contrast, no significant correlation was found between transmigrating CD8^+^ T cells and PSGL-1 expression, illustrating differences between PSGL-1 on CD4^+^ and CD8^+^ T cells. It should be noted that while many of the migration studies involving PSGL-1 show significant decreases in leukocyte adhesion and migration when PSGL-1 is blocked, leukocyte rolling and recruitment is not abolished and can be facilitated by other ligands and adhesive molecules.

Beyond promoting leukocyte migration during inflammatory responses, PSGL-1 also plays a role in the migration of leukocytes during immune homeostasis. Parabiotic mouse experiments have shown that mAb blockade of PSGL-1 results in decreased naive and central memory lymphocyte homing to secondary lymphoid organs (83). PSGL-1 directs this homing behavior through interactions with the chemokines CCL19 and CCL21. Additionally, this homing function of PSGL-1 is lost on activated T cells, as the expression of the core 2–branched sialyl Lewis X on activated PSGL-1 leads to a loss of chemotactic advantage. PSGL-1 also directs the migration of early lymphoid progenitors to the thymus, a step which is necessary for replenishing T cell populations (82). In experiments utilizing adult parabiotic mice, it was shown that this thymic homing by lymphoid progenitors was driven by the binding of PSGL-1 to P-selectin, which is expressed on the thymus endothelium. In addition to facilitating cell homing, PSGL-1 can affect the frequency of circulating myeloid cells. An induced cytopenia model comparing WT and *Selplg^−/−^* mice demonstrated that PSGL-1 constricts release of myeloid precursors from the bone marrow, as well as promoting extravasation of neutrophils and monocytes from blood vessels (146). A wide variety of research has revealed the numerous roles that PSGL-1 plays in adhesion, migration, and in directing the immune response to inflammation. Blockade of the N-terminus provides a possible therapeutic strategy for decreasing leukocyte accumulation in certain inflammatory conditions, however genetic deletion often leads to an opposite outcome, resulting in a worsening of inflammation.

## PSGL-1 Signaling

Much work has been done to uncover the PSGL-1 signaling pathway in neutrophils. Numerous studies have focused on understanding the signaling pathways that are activated upon PSGL-1 engagement. Some of the earliest studies looking into PSGL-1 signaling have demonstrated that engagement of PSGL-1 promotes tyrosine phosphorylation, as well as activation of MAPKs (147). As research progressed, it has become clear there are multiple, complex PSGL-1 signaling pathways in different cell types. While specific proteins and pathways involved in PSGL-1 signaling are still being discovered, careful experimentation has led to an understanding of some of the common signals transduced through PSGL-1. In neutrophils, signaling through PSGL-1 is induced through PSGL-1-selectin interactions. PSGL-1 engagement with P- and E-selectin results in the phosphorylation of the src family kinases (SFKs), Fgr, Lyn, and Hck, as well as Akt, spleen tyrosine kinase (Syk), and phospholipase C (PLC) γ2 (135, 148, 149). This signaling cascade results in lymphocyte function-associated antigen 1 (LFA-1) activation and engagement with intercellular adhesion molecule 1 (ICAM-1), leading to slow rolling in neutrophils. Interestingly, it has been found that L-selectin is vital to this signaling pathway, as *Sell^−/−^* neutrophils failed to phosphorylated SFKs and downstream proteins *in vitro*, and showed increased rolling velocities and diminished adhesion *in vivo* (135).

Specifically, in the context of E-selectin engagement of PSGL-1 on neutrophils, the cytoplasmic domain of PSGL-1 carries out signaling through the src-family kinase Fgr and the ITAM adapters DAP12 and FcRγ (148). While initial results showed that mice lacking Fgr fail to transmit adhesion signals, a follow up study showed that a combined deletion of both Hck and Lyn had a similar result, indicating that while Fgr may be the dominant SFK involved in the PSGL-1-selectin signal transduction, Hck and Lyn together play an important role in this pathway (150). The importance of the ITAM adaptor proteins to PSGL-1 signaling has also been demonstrated. In DAP12 and FCRγ-deficient neutrophils, engagement with E-selectin failed to phosphorylate Syk, and slow rolling was not achieved, indicating the necessity of these two adapter proteins in PSGL-1 driven adhesion signaling. The final steps after Syk recruitment in the E-selectin/PSGL-1-mediated signaling cascade involve the activation of SH2 domain–containing leukocyte phosphoprotein of 76 kD (SLP-76), which in turn activates the Tec kinase Bruton tyrosine kinase (Btk) (150–152). Btk facilitates the phosphorylation of Akt, PLCγ2, and p38 mitogen-activated protein kinase (p38 MAPK), which cumulate in LFA-1-dependent slow rolling of neutrophils on ICAM-1 (149, 153).

PSGL-1 has also been shown to associate with ezrin and moesin. While the interactions of PSGL-1 with SFKs and ITAM adaptor proteins signal to promote slow rolling, it appears that the interactions of PSGL-1 with ezrin and moesin promote leukocyte transcriptional changes and transient MAPK activation. It has been shown that PSGL-1 interacts directly with the amino-terminal domain of both moesin and ezrin, and that these interactions take place in the uropods of activated neutrophils (154, 155). Moesin and ezrin, like DAP12 and FcRγ, are ITAM-adapters that are able to recruit Syk (156). While moesin and ezrin are capable of Syk activation, the signaling outcomes seem to differ from the previously detailed PSGL-1 signaling pathway involving SFKs, DAP12, and FcRγ. *In vitro* experimentation using a leukocyte cell line found that PSGL-1 signaling through ezrin and moesin resulted in an increase in serum response element (SRE) transcription and expression of the early-activation *C-fos* gene. Further *in vitro* experiments showed that the ezrin-radixin-moesin-binding sequence (EBS) on the cytoplasmic tail of PSGL-1 was not necessary for Syk activation (154). While the EBS sequence was shown to support leukocyte tethering to selectins, integrin activation and slow rolling on ICAM-1 is not dependent on ezrin and moesin binding to PSGL-1. Instead, ezrin and moesin engagement with PSGL-1 promotes transient phosphorylation of ERK. From these studies, it is clear that PSGL-1 signaling is multi-faceted and that its engagement with selectins can result in numerous outcomes, ranging from increased activation signals to increased adherence and slow rolling.

There has been less research evaluating PSGL-1 signaling in T cells, however it has been found that PSGL-1 on T cells can have a similar role in signaling integrin-driven adhesion. The use of a PSGL-1 cross-linking antibody resulted in increased LFA-1 clustering (157). This upregulation of LFA-1 promoted adhesion of Th1 cells to ICAM-1, and was driven at least in part by PSGL-1 signaling through PKCα or PKCβII. In addition to promoting adhesion and migration of T cells, PSGL-1 ligation can promote inflammatory responses. Although, many experiments that investigate PSGL-1 inflammatory signaling involve a ligating antibody, these approaches may result in signaling outcomes that differ from PSGL-1 ligand binding. *In vitro* experiments using leukemic Jurkat cells found that antibody ligation of PSGL-1 upregulated transcription of the inflammatory cytokine IL-18 through a pathway involving phosphatidylinositol 3-kinase (PI3K) (81). Antibody ligation of PSGL-1 on Jurkat cells was also found to increase transcription of colony-stimulating factor 1 (CSF-1) in a Syk-dependent manner (158). While these studies show that PSGL-1 can promote inflammatory transcriptional responses, the transcriptional responses peaked at 30 or 60 min, indicating that PSGL-1 inflammatory signals may be transient and require further study.

The question then is raised as to the timing of PSGL-1 signaling, and whether signaling output changes depending on the duration of PSGL-1 engagement and the length of time that a T cell has been activated. While direct mAb engagement of PSGL-1 *in vitro* promoted an increase in inflammatory signals, the timing of PSGL-1 engagement does result in differential signaling outputs. On late stage activated T cells, PSGL-1 signaling has been shown to promote T cell death (159). Both the binding of activated T cells to P- and E-selectin under flow, as well as antibody crosslinking of PSGL-1, can trigger apoptosis in late-stage activated T cells. This PSGL-1-driven apoptosis involves Apoptosis Inducing Factor (AIF) translocation to the nucleus and the subsequent release of cytochrome C, although the full pathway through which PSGL-1 signals induce apoptosis remains to be identified. Further, PSGL-1 signaling has been shown to transduce suppressive signals in periods of prolonged T cell activation. During chronic viral infection, PSGL-1 engagement promotes effector T cell exhaustion (42). While the intracellular signals that direct this PSGL-1 driven enhancement of T cell exhaustion are not known, it has been shown that ligation of PSGL-1 on exhausted CD8^+^ T cells resulted in diminished ERK and AKT signaling (42).

How PSGL-1 signaling in anti-tumor T cells supports their functional exhaustion and inhibitory signaling is not fully known. Since T cells are in an immunosuppressive environment with chronic antigen stimulation inside tumors, PSGL-1 signals may be transient or prolonged depending on ligand binding. In steady-state conditions, PSGL-1 engagement promotes a tolerogenic DC phenotype *in vivo*, increasing the formation of CD4^+^FOXP3^+^ T regulatory cells in the thymus (80). When considering that PSGL-1 signaling prompts immunosuppression both through an increase in the tolerogenic DC phenotype and Treg formation, as well through a decrease in T cell receptor (TCR) signaling, it is clear that targeting PSGL-1 presents a viable path to increase the inflammatory phenotype of CD4^+^ and CD8^+^ T cells. Although PSGL-1 plays a role in signaling for slow rolling and adhesive behavior, this pathway is facilitated by other proteins, as the PSGL-1 genetic deletion does not result in decreased migration of T cells to the tumor site (42). PSGL-1 signaling is complex and much remains to be discovered, but its suppressive signaling in T cells makes it an attractive target for reinvigorating the immune response during cancer.

## Role of PSGL-1 in Disease

When considering PSGL-1 as a therapeutic target, it is necessary to understand the differing roles that PSGL-1 plays depending on the cancer context. As PSGL-1 is known to facilitate attachment and migration, a large body of research has been centered around the role of PSGL-1 in cancer metastasis. In a murine model of multiple myeloma (MM), PSGL-1 on MM cells was shown to interact with P-selectin to promote adhesion signaling and homing of MM cells to the bone marrow (160). In this model, the deletion of PSGL-1 on MM cells led to a significant decrease in tumor initiation and proliferation, illustrating the importance of PSGL-1 in promoting tumorigenesis.

Interestingly, PSGL-1 has also been found on bone-metastatic prostate cancer and lung carcinomas (161, 162). PSGL-1 was linked with metastasis, as it was expressed on a bone-metastatic prostate cancer cell line and in metastatic prostate tumor tissue, indicating that certain cancer types may gain PSGL-1 expression as a part of a metastatic phenotype. The mechanism through which PSGL-1 may facilitate prostate cancer metastasis is unknown, however, in a non-small cell lung cancer (NSCLC) cell line, PSGL-1 was found to facilitate interactions between lung cancer cells and activated platelets (162). This interaction between P-selectin on activated platelets and PSGL-1 on tumor cells is hypothesized to drive metastasis, as activated platelets are known to facilitate metastatic movement of cancer cells (163). In the context of small cell lung cancer (SCLC), cancer cell interactions with P- and E-selectin have been shown to promote robust metastasis. As PSGL-1 is a ligand for both selectins, it is likely that PSGL-1 is involved in the selectin-mediated metastatic behavior of SCLC cells as well (164). Additionally, blockade of P-selectin in mice with gastric cancer decreases metastasis and allows for sustained immune function, a phenotype that PSGL-1 likely plays a role in as the main P-selectin ligand (165). While these experiments show that PSGL-1 plays a pro-metastatic role, the contributions of PSGL-1 on immune cells to this phenotype are still being uncovered. One study showed that PSGL-1 promoted colon cancer metastasis through the recruitment of monocytes to metastatic sites, illustrating how PSGL-1 on immune cells may modulate cancer cell behavior and the TME (166). Although the impact of targeting PSGL-1 specifically on CD4^+^ T cells will have on cancer metastasis is unknown, these studies illustrate that PSGL-1 presents an exciting target for potentially reducing metastatic behavior of tumors.

In addition to promoting cancer metastasis, PSGL-1 is involved in the development of drug resistance, particularly in blood cancers. It has been shown that PSGL-1-mediated interactions between multiple myeloma (MM) cells and macrophages increased ERK1/2 activation, myc upregulation, proliferation, and drug resistance in MM cells (167). The use of a PSGL-1 neutralizing antibody abrogated this MM drug resistance *in vivo*, signifying PSGL-1 as an important driver of MM therapeutic escape. In a separate model of MM, it was found that combination antibody blockade of PSGL-1 and P-selectin lessened bortezomib resistance in MM cells, and led to increased mouse survival (168). Additionally, PSGL-1 was shown to promote chemoresistance in a human acute myeloid leukemia (AML) cell line through interactions with E-selectin (61). Through *in vivo* mouse models of AML, it was seen that *Selplg* ^–/–^ AML blasts showed increased cell cycling, decreased homing to the bone marrow, and increased chemosensitivity. This study showed that PSGL-1 is involved in the formation of bone marrow reservoirs of quiescent, chemoresistant AML cells and is correlated with worse disease outcomes in mice with WT AML blasts.

Taken together, the current body of research has found PSGL-1 to be expressed on numerous human SCLC cells lines (164, 169), on a human alveolar cell carcinoma cell line (162), on human MM cell lines (167), and on a metastatic prostate cancer cell line (161). In the clinic, PSGL-1 expression has been detected in primary acute leukemia cells as well as in some acute lymphoblastic leukemia cells from large patient cohorts (61, 79). Further, a link between disease progression and PSGL-1 expression was shown in a group of MM patients, PSGL-1 was significantly increased in active MM disease when compared to both monoclonal gammopathy of undetermined significance (MGUS) and healthy donors (160). It is clear that PSGL-1 is expressed in many cancers and is involved in disease progression, metastasis, and drug resistance. Importantly, few studies have examined how PSGL-1 expression is regulated in cancers that are not hematopoietic cell-derived. The potential impact of targeting PSGL-1 on tumor control is evident from mouse studies, however the question still remains as to whether PSGL-1 blockade affects CD4^+^ and CD8^+^ T cells and other immune cells within the TME of human cancers.

When investigating the role of PSGL-1 on immune cells in numerous diseases, there is a growing body of literature supporting the notion that PSGL-1 functions as a negative regulator of the immune system. In a murine DSS-induced colitis model, PSGL-1 was shown to decrease the inflammatory immune response, resulting in reduced disease severity (75). There is evidence that in diseases of chronic inflammation, such as systemic lupus erythematosus (SLE), PSGL-1 signaling works to suppress inflammation, as *Selplg^−/−^* mice with SLE suffer more inflammation and early death (170). In this murine SLE model, *Selplg^−/−^* mice increased the amount of the inflammatory chemokine CCL2 present in the kidneys. CCL2 is known to promote cytokine production in CD4^+^ T helper cells, and chemotaxis of T cells and monocytes (171–174). The reduction in CCL2 production driven by PSGL-1 demonstrates another mechanism through which PSGL-1 controls inflammation and limits the induction of T cells responses. Immunotherapeutic blockade of PSGL-1 may increase inflammatory chemokines present *in vivo* and promote a more inflammatory CD4^+^ T cell phenotype.

Mice with genetically deleted PSGL-1 have been valuable in showing the role that PSGL-1 plays in inflammatory immune responses. *Selplg^−/−^* mice have been shown to develop a systemic sclerosis (SSc)-like syndrome. In these mice, the absence of PSGL-1 led to a notable decrease in Tregs in the lungs and an increase in IFN-γ-producing T cells and macrophages, highlighting the role of PSGL-1 in immunosuppression (175). Another autoimmunity study of SSc-like disease in *Selplg^−/−^* mice found increased serum levels of autoantigens, activated DC and CD4^+^ T effector cells in the skin, vascular damage, and increased mortality rates in mice due to loss of PSGL-1 (176). In the absence of PSGL-1, T cells become more inflammatory and can cause chronic inflammation and autoimmunity. The inflammatory T cell phenotype seen in *Selplg^−/−^* mice provides support for the therapeutic targeting of PSGL-1 on CD4^+^ T cells in cancer, as it may provide a way to lessen immune suppression and increase the activation of T cells.

Understanding the differential roles of PSGL-1 on effector T cells and Tregs is particularly important when considering PSGL-1 as an immunotherapeutic target. Sustaining a more effector-like T cell response is vital for the immune system to control cancer, and PSGL-1 can affect the balance of inflammatory and suppressive cells. As mentioned previously, *Selplg^−/−^* mice with DSS-induced colitis show an increased effector T cell to Treg ratio in the colon, a trend that was also observed in the lungs of *Selplg^−/−^* mice (75, 175). In an experimental autoimmune encephalomyelitis (EAE) model, PSGL-1 on Tregs was found to be necessary for suppression of the late stage T cell response (48). Tregs lacking PSGL-1 were unable to limit T cell proliferation and interactions with DCs in the late stages of T cell activation, leading to worsening of the EAE disease phenotype. In addition to limiting the immune response in autoimmune diseases, PSGL-1 on Tregs can affect immune control of cancer (177). Mice lacking P-selectin showed a largely diminished tumor size and a markedly small presence of Tregs in tumors (177). The absence of P-selectin leads to an increase in tumor-infiltrating effector CD8^+^ T cells, an increase in pro-inflammatory cytokines, and a decrease in tumoral TGF-β (177). Although this study did not directly address the role of PSGL-1 as the primary ligand for P-selectin, it is likely supporting P-selectin driven phenotypes. Taken together, it is clear that PSGL-1 promotes development and Treg function and may lead to a reduction in immunosuppression when targeted as an immunotherapy.

Effector T cells are also affected by PSGL-1 signaling. In an *in vitro* setting, stimulated T cell proliferation was negatively regulated by PSGL-1 (43). *In vivo*, PSGL-1-mediated suppression of effector T cell functions has been seen in multiple disease models. In mice with T cell driven inflammatory bowel disease, deletion of PSGL-1 on T cells caused a significant worsening of the disease. The absence of PSGL-1 in mice with a chronic infection led to much more functional, effector-like T cells (42). Further, mice lacking PSGL-1 showed increased melanoma tumor control and reduced T cell exhaustion within the tumor environment (42). Although the mechanism is still being studied, it has been shown that PSGL-1 can dampen TCR signals and effector functions. The work done to understand the roles of PSGL-1 in disease has shown that PSGL-1 can function as a potent suppressor of immune responses. Targeting PSGL-1 on CD4^+^ T cells may be a new opportunity to not only increase effector T cells, but also to reduce the detrimental presence of Tregs in the TME.

## Strategies to Block PSGL-1 on CD4^+^ T Cells in Tumors

**Figure 4:**
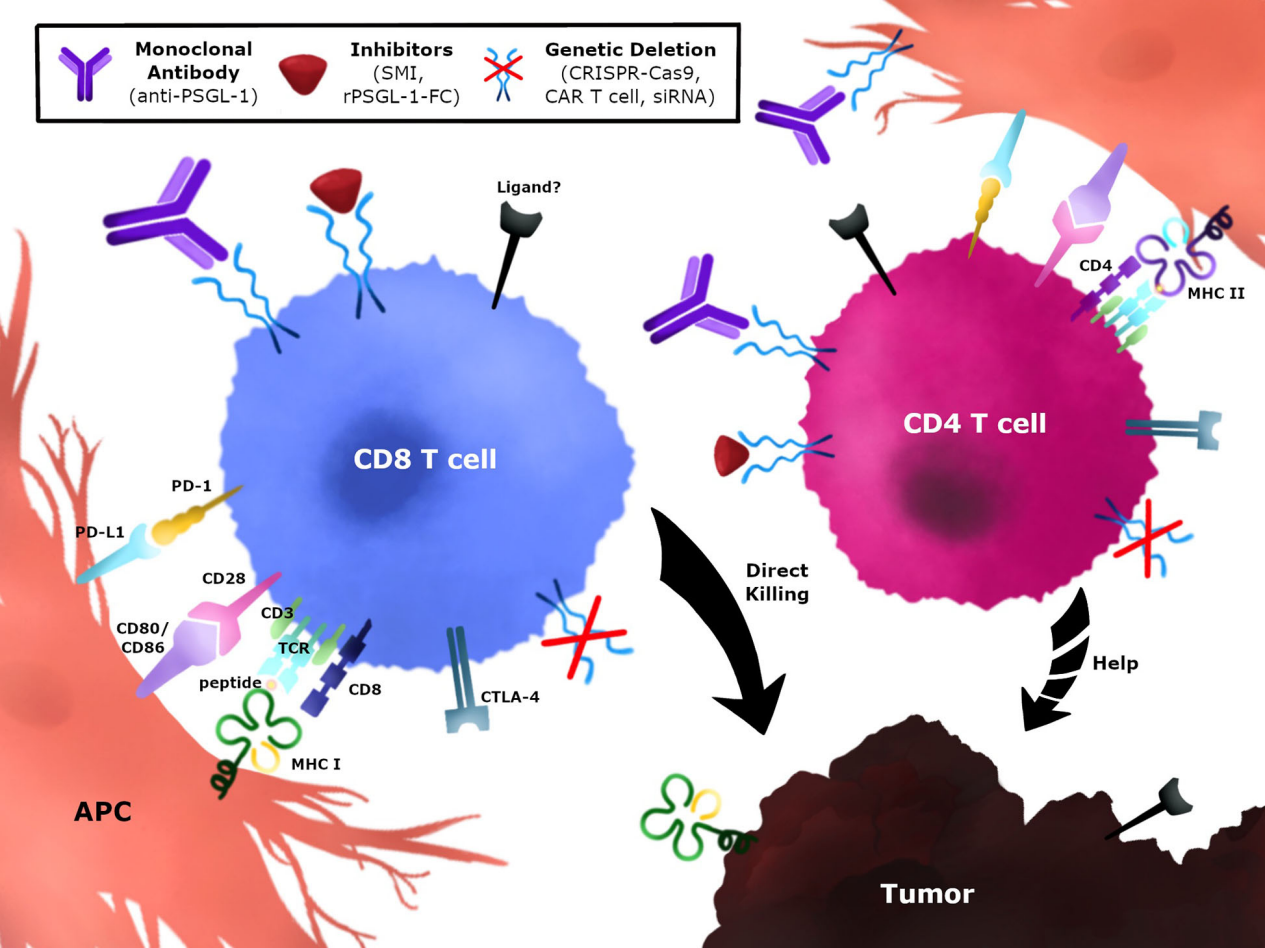
Immunotherapeutic strategies to modulate PSGL-1 in anti-tumor T cells. PSGL-1 can be targeted to modulate T cells in the TME. CD4^+^ and CD8^+^ T cells express PSGL-1 which may be blocked using monoclonal antibodies, small molecule inhibitors, or recombinant PSGL-1 fusion proteins. Blocking PSGL-1 on CD4^+^ T cells could promote helper functions by targeting the tumor directly, providing help for CD8^+^ T cells, or changing the TME. Genetic approaches could target TILs or CAR T cells which could be isolated, expanded, and re-injected in tumors. Strategies can target CD4^+^ T cells, CD8^+^ T cells, or both T cell populations.

Several strategies to target PSGL-1 on anti-tumor T cells could be implemented to block this immune checkpoint and promote tumor control (**Figure 4**). One strategy would be to develop monoclonal antibodies (mAbs) against PSGL-1 and select for biological functions that neutralize and/or antagonize PSGL-1 signaling in T cells (**Figure 4**). These antibodies could be designed to target both CD4^+^ T cells and CD8^+^ T cells, and may also be combined with approved immunotherapies such as anti-PD-1 and anti-CTLA-4. In addition, antibodies targeting PSGL-1 ligands could also be used in therapy once these interactions are confirmed in tumors, as has been successfully tested using anti-VISTA antibodies (129, 178). Additional strategies could be implemented through the use of inhibitors to block PSGL-1 (**Figure 4**). These could include small molecule inhibitors that target the PSGL-1 protein or recombinant PSGL-1 fusion proteins (rPSGL-1-Fc) that may function as decoys to PSGL-1 receptors in tumors, thereby preventing PSGL-1 mediated T cell inhibition. Using genetic approaches to remove PSGL-1 inhibition through the use of cellular therapies such as re-injection of expanded tumor-infiltrating lymphocytes, CAR-T cells, or dendritic cell-based cancer vaccines could be another approach to modulate the tumor microenvironment (**Figure 4**). Targeting PSGL-1 on CD4^+^ T cells could be used to treat patients that are unresponsive to PD-1 blockade. Immune checkpoint resistant patients may have defective CD4^+^ T cells, and preclinical studies suggest that removing PSGL-1 on CD4^+^ T cells could provide superior helper functions, including IL-2 production, to augment the CTL response (42). These therapeutic strategies could be utilized as monotherapies or combination therapies to improve outcomes in cancer patients.

To date, there are a limited number of clinical trials involving PSGL-1. A few trials investigated the use of a recombinant P-selectin glycoprotein ligand-Ig (YSPSL) in liver transplantation (NCT00876902, NCT00450398) and in studies on delayed graft function (179, 180). While YSPSL functions to block the binding of PSGL-1 to selectins, it should be noted that YSPSL is not acting on PSGL-1 directly, and therefore PSGL-1 is still able to interact with its other ligands. Studies that look at directly targeting PSGL-1 with a monoclonal antibody (SelK2) are focused on blood clot prevention (NCT03812328) and airway responses after allergen challenge (NCT04540042). While there are currently no clinical trials investigating PSGL-1 blockade in cancer, there are numerous studies focused on the blockade of VISTA, the recently established ligand for PSGL-1 (129). CA-170, a small molecule inhibitor of both PD-L1 and VISTA, has been tested as a therapeutic for patients with advanced tumors and lymphomas (NCT02812875), as well as metastatic prostate cancer (NCT01288911) (181). An antibody against VISTA (W0180) is currently being tested in combination with Pembrolizumab to treat advanced solid tumors (NCT04564417). Given the positive outcomes of targeting VISTA in pre-clinical trials (182), and considering that PSGL-1 has been established as a binding partner for VISTA and as an immune checkpoint molecule, PSGL-1 should be considered as a potential therapeutic target for cancer in human clinical trials.

## Discussion and Concluding Remarks

PSGL-1 is a highly dynamic molecule expressed on the surface of many innate and adaptive immune cells involved in a variety of diseases, including cancer. PSGL-1 is expressed at high levels on CD4^+^ and CD8^+^ T cells and post-translational modifications add to the complex receptor ligand-pair interactions that occur during T cell immune responses. While the main ligands associated with PSGL-1 include the selectins which mediate trafficking and cell migration, others have recently been described. These include the chemokines CCL19 and CCL21, Siglec-5, versican, and VISTA. These ligands all have the ability to bind PSGL-1 on T cells, and potentially contribute to inhibitory signaling pathways that dampen TCR signals to induce T cell exhaustion in tumors, but more research in the biology of PSGL-1 in tumors is necessary. There are only a few preclinical studies examining the role of PSGL-1 in tumors. One revealed that mice lacking PSGL-1 expression can mount a potent CD4^+^ and CD8^+^ anti-tumor T cell responses in melanoma without impacting their ability to infiltrate in tumors (42). Adoptive cell transfer of *Selplg^-/-^* antigen-specific CD8^+^ T cells in melanoma tumor-bearing mice also improved melanoma tumor control (42). Improved efficacy has already been demonstrated by targeting VISTA, a newly discovered PSGL-1 ligand (129). Since current immune checkpoint therapies are ineffective in certain patients, perhaps defective CD4^+^ T cell help in these patients could be boosted and improved by targeting PSGL-1 on those cells. It is possible that targeting the PSGL-1 immune checkpoint on CD4^+^ T cells could augment the defective CD8^+^ T cell response and improve outcomes in patients resistant to anti-PD-1/anti-CTLA-4 immunotherapies. Another possibility is combination strategies where PSGL-1 is blocked along with anti-PD-1/PD-L1 and/or anti-CTLA-4. Targeting PSGL-1 presents a new and exciting approach to bolstering the anti-tumor T cell response and expanding the immune checkpoint inhibitor toolbox.

## Author Contributions

JMD, KMV, ENN, and RT co-wrote the manuscript and conceived the figures. All authors contributed to the article and approved the submitted version.

## Funding

This work was supported by the National Institutes of Health (R01 AI137239 to RT), Department of Defense (W81XWH-18-1-0738 to RT), The Melanoma Research Alliance (571135 to RT), and in part by (American Cancer Society Institutional Research Grant IRG-16-187-13 to RT) from the American Cancer Society, National Institutes of Health/National Cancer Institute Institutional Training Grant Fellowship in Cancer Biology and Therapeutics (T32 CA009054 to JMD), NIH IMSD training grant (GM055246 to KMV), and T32 virus-host interactions: a multi-scale training program (T32AI007319 to ENN). Funding for graphics was supported by UCI Office of Research and UCI BioSci and Amy Takahashi generated the figures.

## Conflict of Interest

The authors declare that the research was conducted in the absence of any commercial or financial relationships that could be construed as a potential conflict of interest.
